# Epidemiology and patho-anatomical pattern of 2,011 humeral fractures: data from the Swedish Fracture Register

**DOI:** 10.1186/s12891-016-1009-8

**Published:** 2016-04-12

**Authors:** Carl Bergdahl, Carl Ekholm, David Wennergren, Filip Nilsson, Michael Möller

**Affiliations:** Department of Orthopaedics, Sahlgrenska University Hospital Gothenburg/Mölndal, SE-431 80 Mölndal, Sweden

**Keywords:** Population-based epidemiology, Proximal, shaft and distal humeral fracture, Fracture register, Incidence, AO/OTA classification

## Abstract

**Background:**

Humeral fractures are common, but the association between the patho-anatomical fracture pattern and patient characteristics has been inadequately studied and epidemiological knowledge is scarce. Following the introduction of the Swedish Fracture Register (SFR), risk factors for various fractures can be studied, as well as the outcome of different treatments. The objective of this study was to analyse adult humeral fractures in Gothenburg from a descriptive epidemiological perspective.

**Methods:**

All humeral fractures registered in the SFR at Sahlgrenska University Hospital in 2011–2013 in patients aged ≥ 16 years were included. The fractures were divided into humeral segments (proximal, shaft and distal humerus) and analysed according to patient characteristics and patho-anatomical pattern. Furthermore, overall and age-specific incidence rates were calculated.

**Results:**

A total of 2,011 humeral fractures were registered in the SFR, of which 79 % were proximal, 13 % shaft and 8 % distal humeral fractures. The mean age was 66.8 years and women ran a higher risk of humeral fractures than men (female/male ratio 2.4:1). On average, women were older than men at the time of fracture (mean age 70.1 years for women vs. 58.9 years for men). The overall incidence of humeral fractures was 104.7 per 100,000 inhabitants per year, with a segment-specific incidence of 83.0 for proximal fractures, 13.4 for shaft fractures and 8.3 per 100,000 person-years for distal fractures. There was a distinct increase in the age-specific incidence from the fifth decade and onwards, regardless of fracture site. Most fractures occurred in older patients (83 % > 50 years) as a result of a simple or an unspecified fall (79 % > 50 years). Only 1.2 % of all fractures were open injuries and 1.3 % were pathological.

**Conclusion:**

This population-based study provides updated epidemiological data on humeral fractures in a Western-European setting. Most humeral fractures occur as the result of low-energy falls in the elderly population, indicating the influence of age-related risk factors in these fractures. The SFR will be a useful tool for providing continuous information on fracture epidemiology, risk factors and treatment outcome and these population-based data are essential in the planning of future fracture prevention and management.

## Background

Humeral fractures are common in the general population. They comprise approximately 7–8 % of all adult fractures in the western world and their incidence has been reported to increase with age [1]. The treatment of humeral fractures, especially proximal and distal, is controversial and among the most debated of all fracture treatments [2, 3]. With an ageing population and the continuous development of new implants, updated epidemiological data are essential in planning for future fracture management.

There are some previous studies on fracture epidemiology of humeral fractures [1, 3–9]. However, to demonstrate the true epidemiology of a fracture, a study has to include all the fractures occurring in a defined population during a specific time period, and to the best of our knowledge only four fairly updated studies of humeral fractures meet these criteria. One study [1, 8] considers proximal fractures, two diaphyseal fractures [7, 9], while another considers distal fractures [3]. No previous study includes fractures affecting all segments of the humerus.

The Swedish Fracture Register (SFR) was introduced in 2011 at Sahlgrenska University Hospital in order to acquire an improved understanding of the epidemiology and treatment outcome of fractures [10]. The goal for the register is to obtain national coverage. Sahlgrenska University Hospital is the sole provider of fracture care in the city of Gothenburg. All humeral fractures have been registered prospectively since the introduction of the SFR in 2011 and the completeness has been found to be close to 90 % in Gothenburg when compared with official health statistics (personal communication, Nilsson F). The aim of this study was to report on epidemiological data for humeral fractures in Gothenburg in 2011–2013.

## Methods

All data forming the basis of this study were gathered from the SFR. Starting on 1 January 2011 at Sahlgrenska University Hospital in Gothenburg, humeral and tibial fractures were the first fractures registered in the SFR, in patients aged 16 years and older. The registration of fractures in other sites of the body started in 2012. Registration is performed in a web-based program by the treating orthopaedic surgeon, who meets the patient in the emergency department. All in- and out patients are therefore registered prospectively and an increasing number of Swedish hospitals are joining the SFR. At the beginning of 2015, half the departments treating fractures in Sweden had joined the SFR. Work is ongoing to obtain national coverage, as well as full completeness regarding all ages and fractures.

The following information was gathered from the SFR: gender, age, side of fracture, date of the injury and mechanism of injury and fracture classification. The treatment given and the patient-reported outcome were also collected but will not be included in this study.

The mechanisms of injury were divided into six categories; simple fall, fall from a height, unspecified fall, traffic-related, miscellaneous injuries and pathological fractures. A simple fall was defined as a fall from standing height, a fall from height as one from a higher level, such as from furniture or down a stair. An unspecified fall was a fall that was not classified further when registered in the SFR, meaning that it could be either a simple fall or a fall from height. Miscellaneous injuries included all fractures with a mechanism of injury that did not match the other categories, such as fractures sustained in fights, from falling objects or sports-related.

**Fig. 1:**
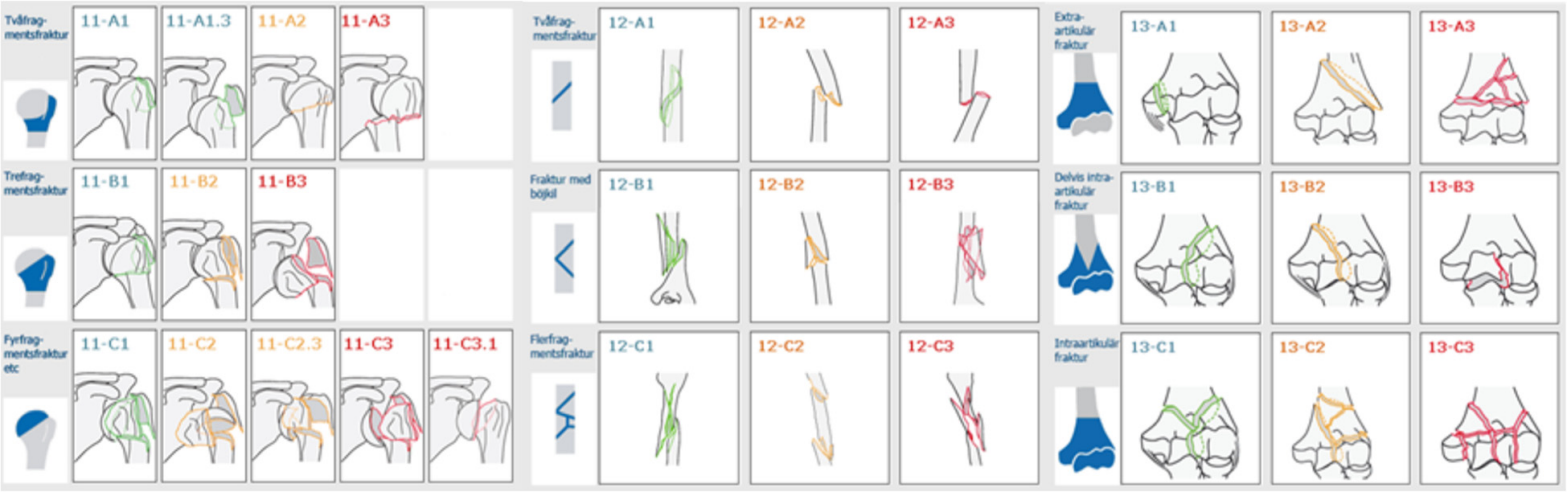
Schematic images of the AO/OTA-classification system of humeral fractures divided into humeral segment, fracture type and group. In the SFR, an additional three subtypes can be selected for proximal fractures, but in this study we have used the nine groups

All fractures were classified radiologically according to the Muller AO/OTA-classification system [11]. Standard radiographs included antero-posterior, lateral and modified axial view for proximal fractures, antero-posterior and lateral for diaphyseal fractures and antero-posterior, lateral and oblique views for distal fractures. In addition, a CT scan was performed if the registrating orthopaedic surgeon regarded it necessary in order to plan for the treatment of the fracture. An AO/OTA-classification manual with schematic images of the different fracture groups and written explanations accompanying the images was used in the on-line classification process in the SFR (Fig. 1). Fractures were divided into proximal, diaphyseal and distal and further subdivided into types and groups, creating a total of nine groups per fracture site. In the SFR, three subtypes (A1.3, C2.3 and C3.1) are also available for proximal fractures, but, to be able to compare the results, these subtypes were included in their respective group (A1, C2 and C3 respectively) in this study.

Pathological, implant-related or periprosthetic fractures were classified in addition to the AO/OTA classification.

By using population data from Statistics Sweden [12], overall and age-specific incidence rates were calculated. As Sahlgrenska University Hospital is responsible for all fracture treatment in Gothenburg, this represents a consecutive series in a defined population of about 632,000 inhabitants (1,895,952 person-years) 16 years of age or older.

## Results

During the three-year study period, a total of 2,011 humeral fractures occurred in 1,986 patients (25 patients had humeral fractures occurring twice during the study period and there were no bilateral fractures). The mean age was 66.8 years (range 16–103) and women were more commonly affected than men (female/male ratio 2.4:1). The age at which fractures occurred differed between women and men, with a mean age of 70.1 years (range 17–103) for women vs. 58.9 years (range 16–96) for men. In both genders, the proximal humerus was by far the most common fracture site (79 %; 1,582/2,011), followed by shaft fractures (13 %; 262/2,011) and distal fractures (8 %; 167/2,011) (Table 1).

**Table 1:** Basic characteristics of all humeral fractures, divided into segments

		Proximal	Shaft	Distal	Total
Gender	Male, N (%)	422 (27)	121 (46)	47 (28)	590 (29)
	Female, N (%)	1160 (73)	141 (54)	120 (72)	1421 (71)
	Total, N (%)	1582 (100)	262 (100)	167 (100)	2011 (100)
Age, yrs	≤50, N (%)	216 (14)	83 (32)	35 (21)	334 (17)
	>50, N (%)	1366 (86)	179 (68)	132 (79)	1677 (83)
Side	Left, N (%)	849 (54)	132 (50)	96 (57)	1077 (54)
	Right, N (%)	733 (46)	130 (50)	71 (43)	934 (46)
Fracture	Open, N (%)	3 (0.2)	6 (2.3)	16 (9.6)	25 (1.2)
	Pathological, N (%)	5 (0.3)	19 (7.3)	3 (1.8)	27 (1.3)
Mean age	Male, yrs (range)	61.8 (16–95)	49.5 (16–91)	57.7 (16–96)	58.9 (16–96)
	Female, yrs (range)	70.5 (18–103)	68.1 (17–98)	68.4 (18–100)	70.1 (17–103)

*N* number, *yrs* years

**Fig. 2:**
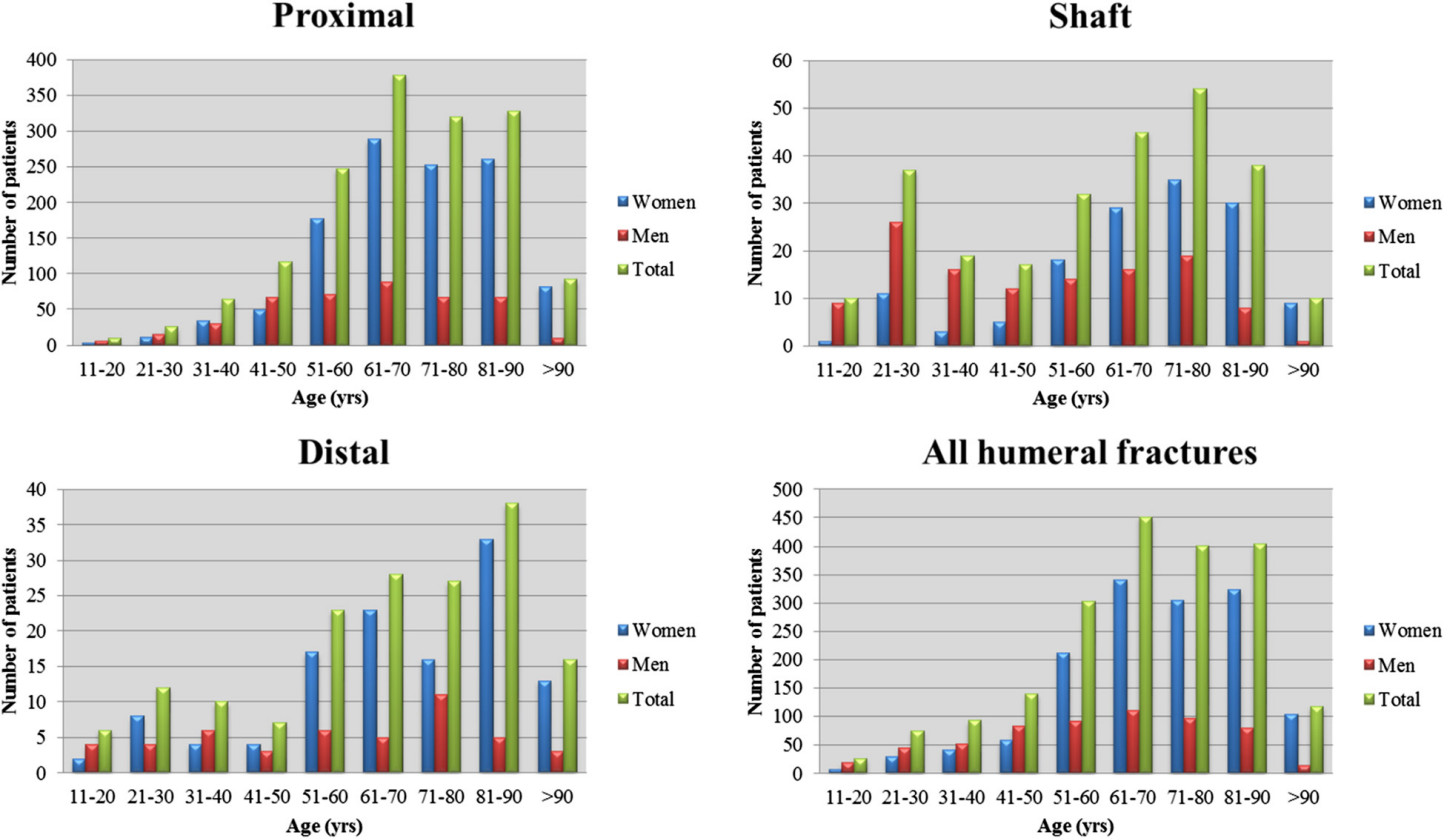
Age and gender distribution per humeral segment and for all 2011 fractures of the humerus

The age and gender distribution of fractures per fracture site (proximal, shaft, and distal) is shown in Fig. 2. The frequency curve per age group was unimodal for proximal and distal fractures, peaking in 61 to 70 and 81 to 90 year olds respectively, whereas it was bimodal for shaft fractures, with a minor peak in the third decade of life and a high peak in the seventh decade of life. The high peak at older ages was particularly evident among women, whereas men showed a more even fracture frequency throughout life, although a slight peak was observed for shaft fractures in men aged 21–30 years. The majority of fractures in patients 50 years or younger occurred in men (59 %; 198/334), whereas the majority of fractures in patients over 50 years occurred in women (77 %; 1,285/1,677).

**Fig. 3:**
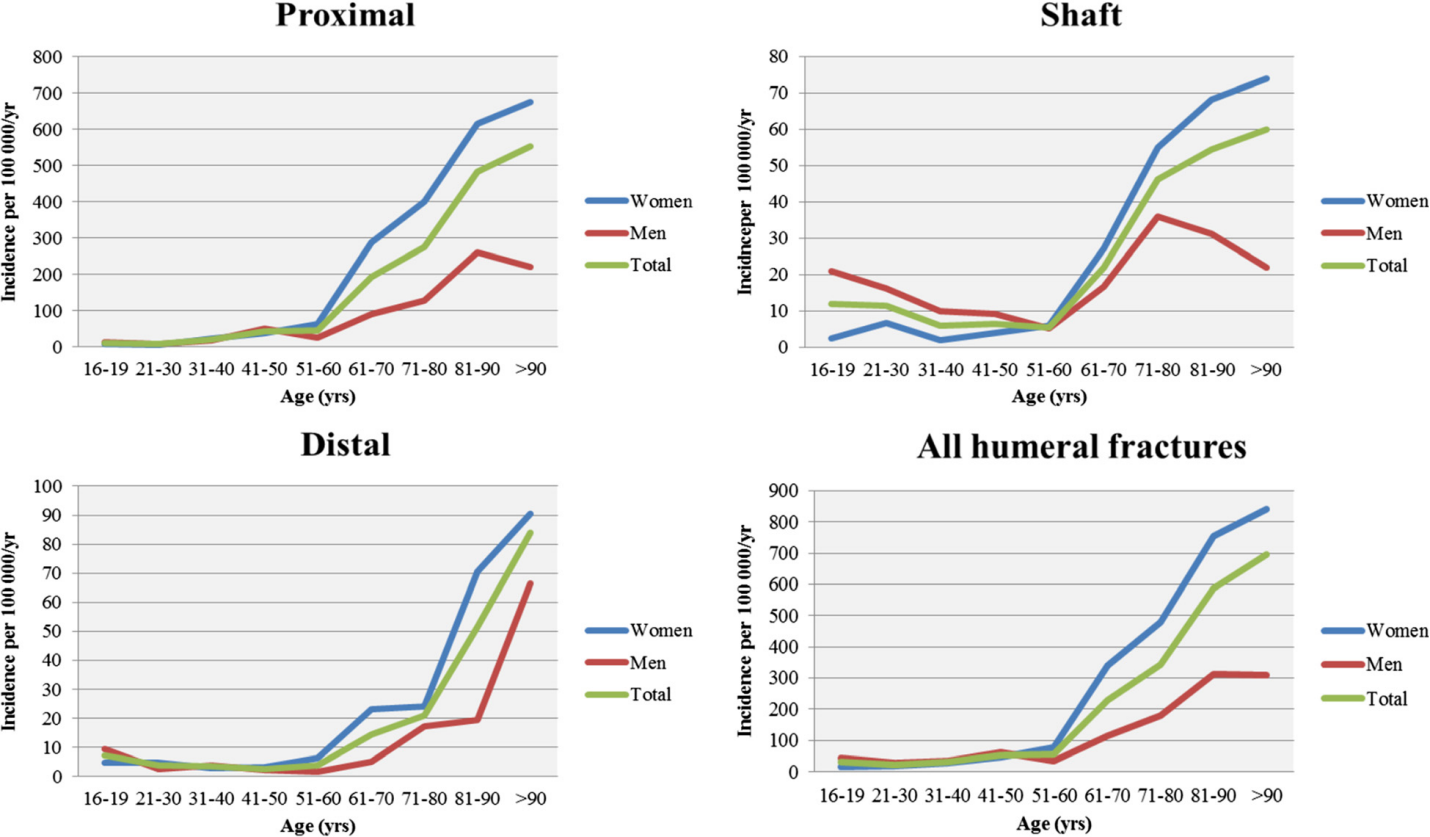
Age- and gender-specific incidence rates of humeral fractures, per humeral segment and total

The overall incidence of humeral fractures was 104.7 per 100,000 inhabitants per year. The highest rate was observed for proximal fractures (83.0 per 100,000 person-years), followed by shaft and distal fractures (13.4 and 8.3 per 100,000 person-years respectively). The age-specific incidence rates of humeral fractures by gender and fracture site are shown in Fig. 3. A distinct increase in overall incidence was observed from the fifth decade and onwards. The increase in incidence with advancing age was greater among women than men. For proximal and shaft fractures, there was a minor decrease in incidence amongst the oldest men, which was not seen in women.

**Fig. 4:**
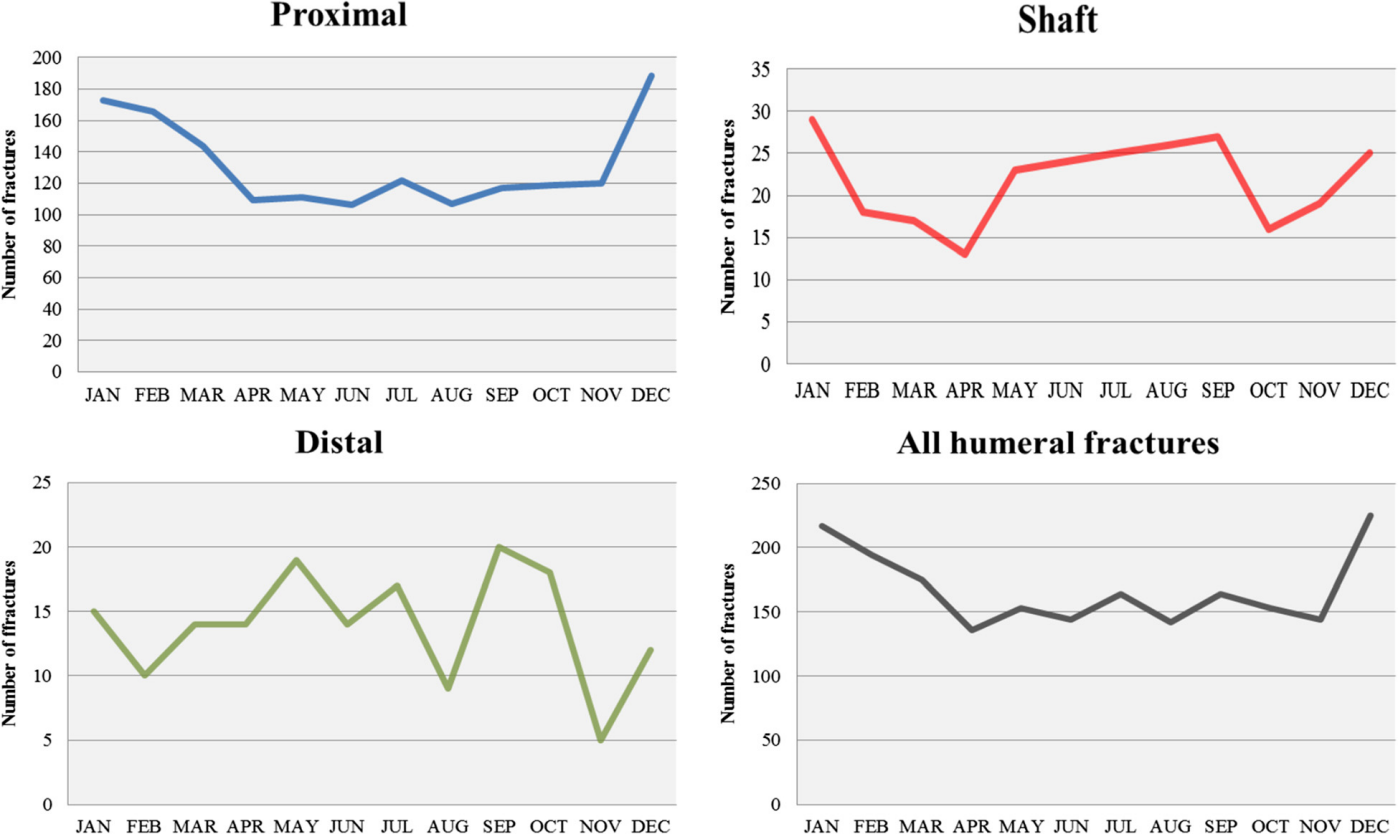
Seasonal variation per humeral segment and for all humeral fractures

The incidence of humeral fractures varied according to the season of the year (Fig. 4). Proximal fractures were more common during the winter months (December-February), whereas shaft fractures peaked during both the winter (December-February) and summer months (May-September). For distal humeral fractures, there was no clear association between the frequency of fractures occurring and the time of year.

Concurrent fractures occurred in 39 of the 1,986 patients with a humeral fracture. Of the associated fractures, the proximal femur was the most common fracture site (21/39), followed by the distal radius (5/39). One patient had two associated fractures: one proximal femoral fracture and one distal radial fracture.

The AO/OTA classification of fractures is shown in Table 2. For proximal humeral fractures, types A (two parts) and B (three parts) were equally common and comprised 44.9 % and 44.0 % respectively. Only 11.1 % were classified as type C. The most common AO/OTA group in the proximal segment was the B1 group (non-displaced, three-part fractures, 29.3 %), followed by A2 (surgical neck, 18.0 %) and A1 (greater tuberosity, 16.5 %). In shaft fractures, type A (simple) was by far the most common fracture type (57.6 %), followed by B (wedge, 25.9 %) and C (complex, 16.5 %). The three most common groups of shaft fracture were A1 (simple spiral, 22.0 %), A3 (simple transverse, 20.4 %) and A2 (simple oblique, 15.3 %). The distal fractures had a more even distribution of AO/OTA types, with 38.6 % A (extra-articular), 25.3 % B (partially articular) and 35.4 % C (completely articular). The most common group of distal fractures was A2 (simple metaphyseal transcolumn, 22.2 %) followed by C3 (multifragmentary, 18.4 %). Among the proximal fractures, the A1 type was more common in younger patients and decreased with age, as opposed to all other fracture types in that segment. In the shaft segment, type C fractures were uncommon in patients under 50 years of age. Instead, the frequency increased with increasing age and most of these fractures affected women. For distal fractures, it was noticeable that the two most common AO/OTA types (A2 and C3) occurred almost exclusively in patients over the age of 50 years. On the other hand, A1 fractures (simple epicondylar) only affected young patients.

**Table 2:** Distribution of all humeral fractures, per segment, into AO/OTA types and groups

	Proximal			Shaft			Distal		
Fracture type	%	Mean age	Women %	%	Mean age	Women %	%	Mean age	Women %
A1	16.5	56.5	61.7	23.3	60.6	63.9	7.8	33.0	53.8
A2	18.0	70.5	74.4	15.3	61.0	52.5	23.4	79.4	76.9
A3	10.4	73.3	66.7	19.8	56.3	38.5	9.0	70.1	73.3
Total A	44.9	66.0	67.9	58.4	59.2	52.3	40.1	68.3	71.6
B1	29.3	69.1	79.0	13.0	53.4	44.1	11.4	61.4	63.2
B2	13.1	71.4	78.8	8.8	58.2	47.8	3.6	63.2	66.7
B3	1.6	65.1	72.0	3.8	50.3	40.0	10.2	58.9	94.1
Total B	44.0	69.6	78.7	25.6	54.6	44.8	25.1	60.7	76.2
C1	3.1	72.4	73.5	8.8	67.1	69.6	6.0	63.5	50.0
C2	6.1	70.9	74.2	2.7	71.9	71.4	10.2	58.6	58.8
C3	1.8	68.3	72.4	4.6	68.5	83.3	18.0	69.6	83.3
Total C	11.1	70.9	73.7	16.0	68.3	73.8	34.1	65.3	70.2

Age and gender distribution, incidence and the AO/OTA type of fracture with their mechanism of injury are shown in Table 3. A similar pattern of sustained mechanisms of injury was reported in all three segments of the humerus. In patients less than 50 years of age, most fractures occurred in men and the predominant mechanism of injury was high-energy trauma (traffic-related injuries, miscellaneous injuries and falls from heights). In patients over the age of 50 years, simple falls were by far the most common mechanism of injury and the overwhelming majority of these patients were women.

**Table 3:** Mechanism of injury, mean age, gender distribution and AO/OTA type of humeral fractures

							AO/OTA type, %		
	Mechanism of injury	N (%)	Open N	Mean age	>50 years, N (%)	Women, %	A	B	C
Prox	Simple fall	932 (59.0)	0	71.0	853 (91.5)	77.7	44.4	43.7	11.9
	Unspecified fall	284 (18.0)	2	69.1	254 (89.4)	73.6	43.3	48.6	8.1
	Fall from height	162 (10.3)	1	68.5	141(87.0)	71.6	40.7	48.8	10.5
	Traffic	89 (5.6)	0	56.6	56 (62.9)	58.4	40.4	48.3	11.2
	Miscellaneous	107 (6.8)	0	49.9	54 (50.5)	50.0	63.6	24.3	12.1
	Pathological	5 (0.3)	0	75.0	5 (100)	60.0	60.0	20.0	20.0
	Total	1579 (100)	3	68.2	1363 (86.3)	73.3	44.9	44.0	11.1
Shaft	Simple fall	107 (41.3)	1	66.4	89 (83.2)	69.2	54.2	28.0	17.8
	Unspecified fall	32 (12.4)	1	68.8	29 (90.6)	59.4	53.1	15.6	31.3
	Fall from height	35 (13.5)	2	63.4	25 (71.4)	54.3	51.4	22.9	25.7
	Traffic	20 (7.7)	1	40.6	4 (20.0)	40.0	65.0	25.0	10.0
	Miscellaneous	46 (17.8)	1	38.2	13 (28.3)	32.6	58.7	37.0	4.3
	Pathological	19 (7.3)	0	71.5	18 (94.7)	26.3	100	0	0
	Total	259 (100)	6	59.5	178 (68.7)	53.8	58.4	25.6	16.0
Dist.	Simple fall	93 (55.7)	5	71.3	84 (90.3)	79.6	39.8	31.2	29.0
	Unspecified fall	21 (12.6)	3	73.0	20 (95.2)	81.0	42.9	14.3	42.9
	Fall from height	18 (10.8)	4	59.4	12 (66.7)	68.4	22.2	27.8	50.0
	Traffic	13 (7.8)	3	62.0	9 (69.2)	53.8	38.5	15.4	46.2
	Miscellaneous	19 (11.4)	1	35.2	4 (21.1)	42.1	52.6	15.8	31.6
	Pathological	3 (1.8)	0	71.7	3 (100)	66.7	66.7	0	33.3
	Total	167 (100.1)	16	65.4	132 (79.0)	71.9	40.1	25.1	34.1
All	Simple fall	1132 (56.5)	6	70.6	1026 (90.6)	77.0	45.0	41.2	13.9
	Unspecified fall	337 (16.8)	6	69.3	303 (89.9)	72.7	44.2	43.3	12.5
	Fall from height	215 (10.7)	7	66.9	178 (82.8)	68.4	40.9	42.8	16.3
	Traffic	122 (6.1)	4	54.6	69 (56.6)	54.9	44.3	41.0	14.8
	Miscellaneous	172 (8.6)	2	45.2	71 (41.3)	44.8	61.0	26.7	12.2
	Pathological	27 (1.3)	0	72.2	26 (96.3)	37.0	88.9	3.7	7.4
	Total^a^	2005 (100)	25	66.8	1673 (83.4)	70.7	46.3	40.0	13.6

^a^Data on the mechanism of injury were available in 2,005 fractures. Data on three fractures in the proximal segment and three fractures in the shaft segment were missing

For fractures in the proximal segment, there was no clear correlation between mechanism of injury and AO/OTA type of fracture. However, in the shaft segment, traffic-related injuries resulted in type A fractures in 2/3 of cases but rarely resulted in type C fractures. In the distal segment, almost half of all fractures caused by falls from heights or traffic-related injuries were type C fractures. Apart from this, there was no obvious pattern regarding the mechanism of injury and the AO/OTA type of fracture.

Of all 2,011 humeral fractures, 25 were open injuries. The majority of these open injuries (16 of 25) were associated with a distal humeral fracture, of which 88 % (14 of 16) were AO/OTA type C. Compared with the overall material, open fractures were more commonly a result of high-energy trauma, but there was no difference in gender or age distribution compared with the overall material.

During the studied time period, 27 pathological fractures were registered. Of these, 17 occurred in men and 10 occurred in women, corresponding to 2.9 % of all male fractures and 0.7 % of all female fractures. Pathological fractures were mainly located in the shaft segment and comprised 7.3 % of all shaft fractures and the peak incidence was in the eight decade of life.

## Discussion

Fractures of the humerus are common and constitute a major health issue. In the western world, it is anticipated that the number of humeral fractures will increase in the near future [13, 14]. In addition, the treatment of these fractures is controversial and technically challenging. In order to plan for future health-care efforts, updated epidemiological data are extremely necessary. With the introduction of a new Swedish fracture register, we are now able, for the first time, to present incidence data on humeral fractures occurring in a defined population in the western part of Sweden and to describe the distribution of various fracture types.

### Age, gender, mechanism of injury

In this study, there was a clear unimodal age distribution of patients sustaining proximal and distal fractures, with a major peak in the seventh to ninth decades. Fractures of the humeral shaft had a bimodal age distribution, with a minor peak in the third decade and a second major peak in the eighth decade. A clear dividing line was observed at fifty years of age for all three humeral segments. The majority of fractures occurring in patients under fifty years of age were among men and high-energy mechanisms were the most common cause of injury. However, most humeral fractures occurred in patients aged fifty years or older and the majority of these were in women sustaining fractures due to simple falls. Analyses of our series regarding age, gender and mechanism of injury reveal general agreement with the four comparable studies previously conducted on fractures of the different segments of the humerus, Court-Brown et al. [8] on proximal humeral fractures, Tytherleigh-Strong et al. [7] and Ekholm et al. [9] on shaft fractures and Robinson et al. [3] on distal fractures. This clearly shows that osteoporosis influences the risk of sustaining any of these fractures. In our series, 86 % of all proximal and 79 % of all distal humeral fractures occurred in patients 50 years or older, with the vast majority of these fractures resulting from a simple or unspecified fall (81 % for proximal and 79 % for distal humeral fractures in patients ≥ 50 years). This confirms the general acceptance that adult fractures of the proximal and distal humerus are osteoporosis related [1]. Our findings regarding shaft fractures of the humerus, where 68 % occurred in patients over 50 years of age, following a simple or an unspecified fall in 66 % of those cases, suggests that a large percentage of shaft fractures are also osteoporotic by nature. This needs to be taken into consideration when planning for the treatment of these fractures. Should surgical intervention be considered, fixation may be compromised by weak osteoporotic bone [15].

### Incidence

The overall incidence rate in our study of 104.7 humeral fractures per 100,000 person-years is slightly higher compared with the extrapolated data on 81.7 humeral fractures per 100,000 person-years from a Scottish study from 2000 of all fractures [1]. For proximal and distal fractures, the overall incidence rate in our series was also higher than those reported by Court-Brown [8] and Robinson [3] on the respective humeral segment, but with the same age pattern of gradually increasing incidence rates from the fifth decade and onwards. A comparison of age-specific incidence rates revealed similar incidence numbers up till fifty years of age, but thereafter the increase in incidence with age in our material was two to three times higher for both men and women. In shaft fractures, the age pattern, with a small peak in adolescence and an increasing incidence from the fifth decade and onwards, matched the age pattern reported by Ekholm [9] and Tytherleigh-Strong [7]. Both overall and age- and gender-specific incidence rates were almost identical to those reported from Stockholm [9] but lower than those from Edinburgh, Scotland [7]. However, the increase in incidence from the fifth decade and onwards was proportionally higher in our material compared with the Scottish material [7].

The steep increase in incidence rate after 50 years of age, not just for women but also for men, demonstrates the influence of age-related risk factors for fractures of the entire humerus. These factors include the increasing influence of osteoporosis, with a subsequent general decrease in bone mass and a higher risk of falls in the elderly population [16]. Previous studies [3, 7–9] have not reported such a marked rise in incidence rates with age. Consequently, the projected increasing age of the population [13, 14], together with our finding of an increased frequency of many complex fracture types of the humerus with increasing age, will pose a serious challenge to health care in the future. Logically, it will result in more complex humeral fractures occurring in fragile patients with osteopenic bone. Bahrs et al. [16] reported this in Germany, with a 100 % increase in dislocated (non-impacted/dislocated types B and C fractures according to the AO/OTA classification) proximal humeral fractures between 1997 and 2011. This calls for precautionary measures to prevent fractures from occurring [17, 18] and evidence-based guidelines to select appropriate fractures for surgical treatment.

### Fracture classification

Our findings relating to the distribution of humeral fractures into AO/OTA types and groups were almost identical for shaft and distal humeral fractures to previously published data [3, 7, 9]. For proximal fractures, they differed from what was reported by Court-Brown et al. [8], with a larger percentage of A fractures (66 %) and fewer B and C fractures (27 % and 6 % respectively) in the Scottish material compared with our findings (45 %, 44 % and 11 % respectively). One possible explanation may be that there was a larger percentage of older patients in our series, as A fractures are more common in young patients and B and C fractures become increasingly common with higher age. Another explanation could be that enhanced imaging techniques have made it possible to detect obscure fracture lines and have therefore shifted the classification into more B and C fractures.

### Mechanism of injury and AO/OTA group

In proximal humeral fractures, there was no clear correlation between mechanism of injury and AO/OTA type of fracture. To the best of our knowledge, there is no previous comparable study reporting on this. For distal fractures, our results are in agreement with Robinson et al. [3]. In our series of shaft fractures, high-energy injuries resulted to a large extent in type A fractures but more seldom in type C fractures compared with low-energy injuries. This differs from what was reported by Tytherleigh-Strong et al. [7] who reported a correlation between high-energy trauma and C fractures. The explanation for the different result might be that our material appears to contain fewer multi-trauma patients, illustrated by a smaller percentage of traffic-related fractures (7.7 % compared with 17.1 %) and open fractures in our material (2.3 %, 5.6 %) and a larger percentage of older women who sustain complex shaft fractures after a simple fall due to the changes in bone quality that constitutes osteoporosis.

### Seasonal variation

Proximal fractures of the humerus showed a seasonal variation in frequency. The fact that there were more fractures during the winter months can probably be explained by the increased occurrence of simple falls due to the icy conditions in Gothenburg during the winter. Court-Brown et al. [8] reported a similar finding in Scotland and they revealed that patients sustaining fractures of this kind are most often elderly fit persons, without the need for social support. This could mean that they are not deterred by weather conditions and still go out when it is cold. The incidence of shaft fractures also varied with the season, with peaks in both the winter and summer months. This might be explained by young patients sustaining fractures in the summer months, during outdoor activities, and older patients sustaining fractures from low-energy falls in the winter. No seasonal variation was found regarding distal humeral fractures and we have not found any previous reports on seasonal variations relating to shaft and distal humeral fractures.

### Open and pathological fractures

Open fractures mostly occurred in the distal humeral segment, comprising almost one in ten distal humeral fractures. This was slightly more than the one in fourteen reported by Robinson el al. [3]. We agree with their finding that most of these fractures were AO/OTA type C. Pathological fractures occurred almost exclusively in the shaft segment and accounted for 7.3 % of all humeral shaft fractures in our series. This corresponds well with the findings in Scotland [7] and Stockholm [9] of 8.5 % and 6.2 % respectively.

### Strengths and limitations

This study has a few potential limitations. First, at the time of writing, the registration completeness of humeral fractures in the SFR in Gothenburg is not known. The Swedish health-care system is organised in such a way that all patients with fractures of the humerus are referred to major hospitals. In Gothenburg, all patients with suspected fractures are referred to Sahlgrenska University Hospital for a first assessment at the emergency department. Verified fractures are registered in the SFR as described previously. Subsequently, assigned orthopaedic surgeons at the orthopaedic department review all fractures and verify that they have been registered [10]. We therefore have reason to believe that our series represents most patients who sustained a humeral fracture in Gothenburg between 2011 and 2013 and that our series is representative of the whole population. A study of the completeness of registration of humeral fractures in the SFR in Gothenburg in 2011–2013 is pending (personal communication, Nilsson F).

Second, orthopaedic surgeons with varying experience classify the fractures in the register. Factors shown to affect the inter- and intra-observer reliability are level of experience and number of classification groups [19], with higher accuracy with increasing experience and fewer groups. However, validation data reveal moderate to substantial inter-observer reliability [20] of the classification of humeral (unpublished observation, Stjernström S) and tibial fractures [21] recorded in the SFR (kappa value 0.58 and 0.56 respectively), which is in line with previous studies of fracture classification and show that the classification of fractures in the SFR is reliable.

One obvious strength of this study is that it is based on prospectively collected population data, retrieved from a population register. Although not addressed in this study, information on treatment and outcome following treatment is also available from the register. Since registration is continuously proceeding, the evaluation of trends and variations in incidence and treatment modalities will be possible during the coming years.

## Conclusion

Two thirds of all humeral fractures in a Swedish adult population occur as a result of simple or unspecified falls among people older than 50 years. This indicates that osteoporosis and an increased propensity to fall constitute important risk factors for humeral fractures. The anticipated ageing of the population will therefore most probably increase the number of humeral fractures, a development that will pose a serious challenge to the health-care system. The recently started SFR will provide continuous information about fracture epidemiology, risk factors and treatment outcome. Population-based data of this kind are essential in the planning of future fracture prevention and management.

### Ethics approval and consent to participate

The Central Ethical Review Board, University of Gothenburg (Reference no. 712/14), approved the study.

### Consent for publication

Not applicable.

### Availability of data and materials

The Swedish Fracture Register website is found at www.frakturregistret.se. The log in process and the restriction to use is thoroughly described by Wennergren et al. in 2015 [21].

## Abbreviations

AO: Arbeitsgemeinschaft für Osteosynthesefragen
CT: computed tomography
OTA: Orthopaedic Trauma Association
SFR: Swedish Fracture Register
